# Severe hemolytic exacerbations of Chinese PNH patients infected SARS‐CoV‐2 Omicron

**DOI:** 10.1002/iid3.966

**Published:** 2023-08-08

**Authors:** Hui Yang, Xingxing Chai, Yuemin Gong, Xinyu Zhang, Lingling Wang, Xin Zhou, Xiaoyu Chen, Jinge Xu, Dan Xu, Guangsheng He, Jianyong Li

**Affiliations:** ^1^ Key Laboratory of Hematology of Nanjing Medical University, Department of Hematology Collaborative Innovation Center for Cancer Personalize, The First Affiliated Hospital of Nanjing Medical University, Jiangsu Province Hospital Nanjing Jiangsu China; ^2^ Department of Hematology The Second People's Hospital of Lianyungang Lianyungang Jiangsu China; ^3^ Department of Hematology Wuxi People's Hospital Affiliated to Nanjing Medical University Wuxi Jiangsu China; ^4^ Department of Hematology The Second Affiliated Hospital of Xuzhou Medical University Xuzhou Jiangsu China; ^5^ Department of Hematology Funing People's Hospital Yancheng Jiangsu China

**Keywords:** complement inhibitor therapy, COVID‐19, hemolysis, Omicron, paroxysmal nocturnal hemoglobinuria

## Abstract

**Introduction:**

Paroxysmal nocturnal hemoglobinuria (PNH) is characterized by hemolytic anemia, bone marrow failure, thrombophilia. COVID‐19, caused by a novel severe acute respiratory syndrome coronavirus 2 (SARS‐CoV‐2) with many variants including Omicron.

**Methods:**

This study collected demographic and clinical data of 20 PNH patients with SARS‐CoV‐2 Omicron infection.

**Results:**

They all were with high disease activity, and LDH level exceeded any documented since the diagnosis of PNH, and those reported in the literature for previously stable treatment with complement inhibitors. D‐dimer level elevated in 10 patients. 2 patients developed mild pulmonary artery hypertension. Glomerular filtration rate declined in 5 patients. 1 patient developed acute renal failure and underwent hemodialysis. Anemia and hemolysis were improved in 5 patients treated with eculizumab.

**Conclusions:**

Hemolytic exacerbation of PNH with COVID‐19 is severe and eculizumab may be an effective treatment.

## Graphical abstract

Severe acute respiratory syndrome coronavirus 2 infection could induce a hemolytic exacerbation in patients with paroxysmal nocturnal hemoglobinuria but eculizumab can effectively control acute hemolysis.

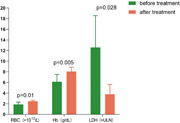

## 1 INTRODUCTION

Paroxysmal nocturnal hemoglobinuria (PNH) is an acquired and clonal disease characterized by hemolytic anemia, bone marrow failure, thrombophilia, and multiorgan damage, which results from the mutation of the *X*‐linked *PIGA* gene. The blockade of glycosylphosphatidylinositol (GPI) synthesis caused by mutation results in the absence of GPI‐anchored protein (such as CD55 and CD59), and CD55‐deficient and CD59‐deficient blood cells are more susceptible to complement attack and lysis.^1^, ^2^ Coronavirus disease 2019 (COVID‐2019) first emerged in Wuhan, Hubei, China, by severe acute respiratory syndrome coronavirus 2 (SARS‐CoV‐2).^3^ SARS‐CoV‐2 was reported multiple variants, including Alpha, Beta, Gamma, Delta, Omicron, and Omicron shows a 13‐fold increase in viral infectivity than Delta variant.^4^ SARS‐CoV‐2 activate the complement system through the classical pathway, lectin pathway, and the alternative pathway. Specific antibody directed against the receptor‐binding domain of the spike protein initiates the classical pathway, the binding of mannose‐binding lectin with SARS‐CoV‐2 spike protein triggers the lectin pathway and SARS‐CoV‐2 spike protein may dysregulate the alternative pathway by binding heparan sulfate and competing with factor H, which is a negative regulator of complement activity.^5^, ^6^ After SARS‐CoV‐2 infection, patients with PNH more likely suffered the hemolysis,^7^, ^8^, ^9^, ^10^, ^11^, ^12^, ^13^, ^14^, ^15^, ^16^ patients usually presented visible hemoglobinuria and a small number patients showed pancytopenia.^17^ Terminal complement inhibitors (such as eculizumab) are promising in COVID‐19 treatment by blocking the formation of membrane attack complex and reducing proinflammatory and prothrombotic influence.^5^, ^6^, ^18^ Due to the rarity of PNH, the clinical features of patients infected with SARS‐CoV‐2 were mostly case reports. China has been heavily affected by the SARS‐CoV‐2 Omicron outbreak with the peak in mid‐December 2022. We collected clinical data from 20 PNH patients infected with SARS‐CoV‐2 Omicron and treated 5 patients with eculizumab.

## 2 PATIENTS AND METHODS

### 2.1 Patients

The study reviewed clinical data of all outpatients in the four centers: the First Affiliated Hospital with Nanjing Medical University (Nanjing, China), the second people's hospital of Lianyungang (Lianyungang, China), Wuxi People's Hospital (Wuxi, China), and Funing People's Hospital (Funing, China) from December 2022 to February 2023. Twenty patients with PNH infected SARS‐CoV‐2 Omicron were enrolled and registered in this study. Patients signed informed consent. The study was conducted according the Declaration of Helsinki and approved by the hospital ethics committee.

### 2.2 Diagnostic criteria

PNH patients were confirmed by flow cytometry using a reagent called fluorescent aerolysin (FLAER), which is the principal virulence factor of the bacterium Aeromonas hydrophilia, binds selectively and with high affinity to the GPI anchor.^2^

Patients infected with COVID‐19 were considered to have fever, cough, nasal congestion, sore throat, or other uncomfortable symptom and their reverse transcription polymerase chain reaction or antigen testing (by colloidal gold method) for SARS‐CoV‐2 was positive.^3^

High disease activity (HDA) was defined as evidence of hemolysis (elevated lactate dehydrogenase [LDH] ≥1.5 times upper limit of normal [ULN]) and a history of at least one of the following signs or symptoms—fatigue, hemoglobinuria, abdominal pain, dyspnea, anemia (hemoglobin <100 g/L), MAVEs (including TEs), dysphagia, or erectile dysfunction.^19^

### 2.3 Methods

We collected the patient demographics, clinical manifestations, laboratory data^2^, ^3^ for understanding degree of anemia, hemolysis, thrombosis, organ damage, (including complete blood count [CBC], LDH, total bilirubin [TBIL], indirect bilirubin [IBIL], creatinine, glomerular filtration rate [GFR], blood urea nitrogen [BUN], d‐dimer, IL‐6, and pulmonary artery systolic pressure), treatment, and outcomes in the cohort. Data on 20 patients with PNH registered in four centers is to evaluate the occurrence and clinical characteristics of SARS‐CoV‐2 infection, and the efficacy of eculizumab.

### 2.4 Laboratory tests

All laboratory tests were done by the medical centers where the individual patients visited.

CBC used EDTA as anticoagulants by blood cell analyzer following the two principles of electricity (electrical impedance method and radio frequency conductance method) and optical (laser scattering method and spectrophotometry).

LDH, TBIL, IBIL, creatinine, GFR, and BUN used heparin as anticoagulants by a biochemical analyzer following the principle of photoelectric colorimetry.

d‐dimer used sodium citrate as anticoagulants detected by the technique of immunofluorescence.

IL‐6 used EDTA as anticoagulants detected by the technique of chemiluminescence.

### 2.5 Statistics analysis

Categorical variables were described using frequencies and percentages, and continuous variables were described using median, minimum, and maximum. Shapiro–Wilk test is used to test whether the data conform to a normal distribution. The degree of SARS‐CoV‐2‐induced hemolysis compared with the literature was examined by Mann–Whitney *U* test. Paired samples conformed to a normal distribution use paired sample *T*‐test, otherwise Wilcoxon test is used for examination. Data management and analysis were performed using IBM SPSS Statistics, Version 26.0.: IBM Corp. *p* Values .05 was considered statistically significant.

## 3 RESULTS

### 3.1 Status of hemolytic episodes with SARS‐CoV‐2 Omicron infection

A total of 20 PNH patients diagnosed with SARS‐CoV‐2 Omicron infection were enrolled in this study. Six (30%, 6/20) were male, 14 (70%, 14/20) were female and the median age was 37 years (range: 15−75 years). Eleven patients (55%, 11/20) presented classical PNH and 9 patients (45%, 9/20) were diagnosed with PNH/AA. They accepted glucocorticoid maintenance and transfusion in the past. None of them were treated with complement inhibitors. The median time to episode occurred is 3 days after SARS‐CoV‐2 Omicron infection (1−5 days).

Ten patients (50%, 10/20) complained of hemoglobinuria. Other 10 patients (50%, 10/20) presented with worsen fatigue and cytopenia. Although no thrombotic event happened, d‐dimer (median 2.55 mg/L, range: 0.57−4.47 mg/L) was elevated in 10 patients, and all of them received prophylactic low molecular weight heparin.

Mild pulmonary hypertension was found in 2 patients (32 and 35 mmHg, respectively). The GFR in 5 patients (25%, 5/20) was lower than 60 mL/min/1.73 m.^2^ One patient underwent hemodialysis due to acute renal failure.

We compared the current Omicron‐induced hemolysis and previous most drastic hemolysis. Five (29.41%, 5/17) were male, 12 (70.59%, 12/17) were female and the median age was 38 years (range: 17−73 years). Ten patients (58.82%, 10/17) presented classical PNH and 7 patients (41.18%, 7/17) were diagnosed with PNH/AA. The level of LDH (2555 vs. 1542 U/L, 9.43 × ULN vs. 5.62 × ULN, *p* = .011), TBIL (49.56 vs. 28.92 μmol/L, *p* = .031), and IBIL (29.31 vs. 17.8 μmol/L, *p* = .017) were higher than the most severe hemolysis previously recorded. Anemia became worsened compared to the previous period—RBC (2.17 × 1012 vs. 2.42 × 1012/L, *p* = .356), Hb (66 vs. 71 g/L, *p* = .459) (Table 1). The decrease in GFR was nearly statistically significant (113.65 vs. 118.3 mL/min/1.73 m,^2^ *p* = .055).

**Table 1:** Comparison of current hemolysis with the most severe hemolysis previously.

	Current Omicron‐induced hemolysis	Previous most drastic hemolysis	*p* Value
WBC (×10^9^/L) (range)	3.11 (1.54−10.54)	3.64 (2.23−9.58)	.796
ANC (×10^9^/L) (range)	2.24 (0.68−9.35)	1.8 (0.5−7.81)	.636
RBC (×10^12^/L) (range)	2.17 (1.14−3.28)	2.42 (1.37−3.27)	.356
Hb (g/L) (range)	66 (38−88)	71 (47−94)	.459
BPC (×10^9^/L) (range)	140 (22−306)	141 (12−310)	.446
LDH (U/L) (range)	2555 (672−8482)	1542 (733−4157)	.011
LDH (×ULN) (range)	9.43 (2.48−31.3)	5.62 (2.7−15.34)	.011
Creatinine (μmol/L) (range)	68 (35.8−186)	62 (38.3−215)	.113
GFR (mL/min/1.73 m^2^) (range)	113.65 (32.44−136.3)	118.3 (27.23−143.59)	.055
BUN (mmol/L) (range)	4.59 (2.4−10.38)	4.76 (2.2−11)	.623
TBIL (μmol/L) (range)	49.56 (11−104.3)	28.92 (12.4−94.1)	.031
IBIL (μmol/L) (range)	29.31 (7.5−84.9)	17.8 (10.1−72.2)	.017
d‐dimer (mg/L) (range)	0.6 (0.31−4.15)	0.59 (0.12−6.76)	.695

Abbreviations: BUN, blood urea nitrogen; GFR, glomerular filtration rate; IBIL, indirect bilirubin; LDH, lactate dehydrogenase; TBIL, total bilirubin; ULN, upper limit of normal.

### 3.2 Efficacy of eculizumab

**Figure 1:**
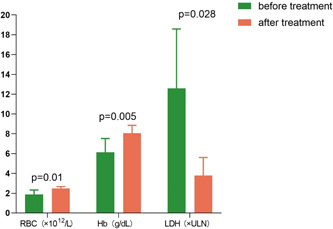
Eculizumab improved the RBC count (1.96 × 10^12^ vs. 2.45 × 10^12^/L, *p* = .01), levels of Hb (64 vs. 83 g/L, *p* = .005), and LDH (13.53 × ULN vs. 3.38 × ULN, *p* = .028) significantly. LDH, lactate dehydrogenase; ULN, upper limit of normal.

Five patients were administrated with eculizumab. One (20%, 1/5) were male, 4 (80%, 4/5) were female and the median age was 59 years (range: 26−67 years). Three patients (60%, 3/5) presented classical PNH and 2 patients (40%, 2/5) were diagnosed with PNH/AA. After eculizumab treatment, hemolysis was controlled (LDH 13.53 × ULN vs. 3.38 × ULN, *p* = .028), and RBC count (1.96 × 10^12^ vs. 2.45 × 10^12^/L, *p* = .01), and level of hemoglobin (64 vs. 83 g/L, *p* = .005) were also improved significantly (Table 2 and Figure 1). The improvement in IBIL was nearly statistically significant (31.71 vs. 20.69 μmol/L, *p* = .057). The elevated d‐dimers in 2 patients also decreased significantly after treatment—4.04 versus 1.47 mg/L, 2.15 versus 1.37 mg/L, respectively. During hemolysis, 1 patient had a high IL‐6 serum concentration, 31.29 times higher than the upper limit of the normal—165.83 pg/mL while after eculizumab treatment IL‐6 drops to 4.98 pg/mL. Because of emergency use, the patients treated by eculizumab could not be vaccinated against *Neisseria meningitidis* and *Streptococcus pneumoniae* in advance, but infection did not occur with the whole course of penicillin prophylaxis.

**Table 2:** Improvement of the hemolysis by eculizumab.

	Before eculizumab therapy	After eculizumab therapy	*p* Value
WBC (×10^9^/L) (range)	2.85 (2.27−10.54)	3.09 (2.1−5.64)	.504
ANC (×10^9^/L) (range)	2.24 (0.74−9.35)	1.79 (0.17−4.62)	.686
RBC (×10^12^/L) (range)	1.96 (1.32−2.47)	2.45 (2.24−2.66)	.01
Hb (g/L) (range)	64 (43−80)	83 (67−87)	.005
BPC (×10^9^/L) (range)	74 (50−306)	81 (38−319)	.448
LDH (U/L) (range)	3667 (1075.1−4887)	915 (460−1822)	.028
GFR (mL/min/1.73 m^2^) (range)	84.47 (36.31−130.54)	85.53 (42.25−129)	.742
IBIL (μmol/L) (range)	31.71 (14.7−83.5)	20.69 (4.3−24.1)	.057

Abbreviations: ANC, absolute neutrophil count; BMD, bone marrow disease; BPC, blood platelet count; BUN, blood urea nitrogen; GFR, glomerular filtration rate; Hb, hemoglobin; IBIL, indirect bilirubin; LDH, lactate dehydrogenase; RBC, red blood cell count; TBIL, total bilirubin; ULN, upper limits of normal; WBC, white blood cell count.

### 3.3 Outcomes of COVID‐19 and PNH

In our study, 12 of 20 (60%) were hospitalized. Two (2/20, 10%) patients developed severe COVID‐19—one with severe viral pneumonia but recovered after two doses of eculizumab with oxygen support and the other with acute renal failure but recovered after hemodialysis.

Of the 5 patients using eculizumab, 2 were not in HDA status. But 13 of the other 15 patients remained in HDA status during the second week after infection with SARS‐CoV‐2 Omicron.

## 4 DISCUSSION

PNH is a complement‐mediated hemolytic anemia^1^ and SARS‐CoV‐2 activates complement and inflammatory factor storm.^6^ PNH patients infected with SARS‐CoV‐2 are more likely to have hemolytic episodes. Twenty patients all had HDA in this study and had severe hemolysis and anemia: LDH, IBIL increased and red blood cell count, hemoglobin declined significantly.

Current study found that Omicron‐induced hemolysis was more intense than ever happened since diagnosis. On the one hand, SARS‐CoV‐2 triggered complement activation through three pathways^5^, ^6^ and on the other hand, coronavirus infection activates monocyte, macrophage, and dendritic cell, then releases IL‐6 and amplifies cytokine cascade.^20^ Under dual attack, it causes severe hemolysis in patients suffering from PNH.

In this study, there was no thromboembolism, but d‐dimer values of a half of patients were beyond the ULN. Zlatko et al.^16^ reported 1 PNH patient who presented with deep vein thrombosis as the first sign of COVID‐19. SARS‐CoV‐2 may increase thrombophilia in PNH in the context of multiple triggers, such as increased inflammatory factors, endothelial injury, platelet, and thrombin activations. Acute kidney injury of PNH will be caused by a variety of reasons, such as the direct toxicity of free hemoglobin released by broken red blood cells, the constriction of renal blood vessels caused by NO consumption, and the direct damage caused by the coronavirus and cytokine storm.^2^, ^21^, ^22^ In our cohort, there were 5 patients with severe renal function decline (GFR ≤60 mL/min/1.73 m^2^), even 1 accepted hemodialysis.

C5 inhibitors have been recommended as the first line treatment for PNH,^23^ greatly improving the poor prognosis of PNH. C5 inhibitors protect PNH clone from attack by blocking the complement activation pathway and significantly improve hemolytic anemia, reduce events of thrombosis, and alleviate damage of renal function.^24^, ^25^, ^26^, ^27^ Meanwhile, eculizumab has been used to treat severe COVID‐19.^28^ Excess C5a induces the release of proinflammatory cytokines from innate immune cells which is thought to play a key role in acute lung injury.^29^ After eculizumab stops the cleavage of C5, the production of C5a is reduced. In an Annane's study^30^ of 80 patients with severe COVID‐19, 35 patients treated with additional eculizumab showed higher survival rate (82.9% vs. 62.2%, *p* = .04) and improved tissue oxygenation compared with 45 patients who received supportive care alone. Another case reported that after Diurno^31^ administrated eculizumab to 4 patients with severe COVID‐19, their inflammatory markers declined and recovery time was cut short. The literature^10^, ^12^, ^13^, ^14^, ^16^ documented less frequent hemolysis following SARS‐CoV‐2 infection in PNH patients who regularly used complement inhibitors. Compared with cases with LDH values reported in the literature, hemolysis in current study was more severe (LDH 7.47 × ULN vs. 2.04 × ULN, *p* < .001). In our study, 5 patients receiving eculizumab achieved improvement in hemolysis and anemia, and COVID‐19 was also relieved. Eculizumab maybe a good choice for patients with PNH after SARS‐CoV‐2 infection.

In conclusion, our study preliminarily demonstrate SARS‐CoV‐2 infection could induce a hemolytic exacerbation in patients with PNH but eculizumab can effectively control acute hemolysis. It is the unavoidable association between PNH hemolytic exacerbation and visiting hospital. A large amount of long‐term follow‐up data are still needed to assess the impact of SARS‐CoV‐2 on patients with PNH, evaluate the dose and duration of eculizumab, and develop better prevention and control plans.

## AUTHOR CONTRIBUTIONS

*Research idea and study design*: Hui Yang and Xingxing Chai. *Data collection*: Hui Yang, Yuemin Gong, Xinyu Zhang, Lingling Wang, Jinge Xu, and Dan Xu. *Data analysis*: Hui Yang, Xingxing Chai, and Xiaoyu Chen. *Wrote the manuscript*: Hui Yang and Xingxing Chai. *Supervision or mentorship*: Xin Zhou, Jianyong Li, and Guangsheng He.

## CONFLICT OF INTEREST STATEMENT

The authors declare no conflict of interest.

## ETHICS STATEMENT

This study was approved by the First Affiliated Hospital of Nanjing Medical University ethics committee (Ethics approval document 2020‐SR‐421) conducted following the principle of the Helsinki Declaration. Informed consent was obtained from all individual participants included in the study. The authors confirms that the work has not been published before and the publication has been approved by all coauthors.

## Data Availability

This article contains all the data generated or analyzed during this study. Any further queries should be directed to the corresponding author.
